# Primary membranoproliferative glomerulonephritis: natural history, pathogenesis, and treatment

**DOI:** 10.3389/fneph.2026.1747678

**Published:** 2026-02-13

**Authors:** Edward J. Filippone, John L. Farber

**Affiliations:** 1 Division of Nephrology, Department of Medicine, Sidney Kimmel Medical College at Thomas Jefferson University, Philadelphia, PA, United States; 2 Department of Pathology, Sidney Kimmel Medical College at Thomas Jefferson University, Philadelphia, PA, United States

**Keywords:** alternate pathway, C3 glomerulonephritis, C3 glomerulopathy, complement system, dense deposit disease, iptacopan, membranoproliferative glomerulonephritis, pegcetacoplan

## Abstract

Primary membranoproliferative glomerulonephritis (MPGN) is an ultrarare disease characterized by immunofluorescence microscopy as either immune-complex mediated (IC-MPGN) or C3 glomerulopathy (C3), the latter subdivided by electron microscopy to C3 glomerulonephritis (C3GN) and dense deposit disease (DDD). Both IC-MPGN and C3G typically have obvious C3 staining differentiating them from other causes of MPGN histology. Secondary causes must be excluded, including infections, autoimmune disease, and neoplasia. Clinical presentations are variable, including urinary sediment abnormalities, nephrotic syndrome, or a rapidly progressive course. The prognosis is unfavorable with about 50% reaching kidney failure by 10 years. Recurrence following transplantation is frequent, and allograft survival is shortened. The pathogenesis involves dysregulation of the alternate pathway (AP) of complement. Possibly 20% of patients harbor pathogenic mutations in AP proteins or their regulators, and up to 80% have autoantibodies impairing normal regulation. Paraproteins are found in 20 – 40% of otherwise primary MPGN, either directly detectable on biopsy (IC-MPGN) or as dysregulators of the AP. Therapy of MPGN begins with supportive care as for all glomerulopathies. Paraproteins require clone-directed therapy. When immunosuppression is considered, complement inhibition should be first line. Two agents are now FDA approved for C3G, the oral Factor B inhibitor iptacopan and the subcutaneous C3-inhibitor pegcetacoplan, the latter also approved for IC-MPGN. If complement inhibition is unavailable, MMF/steroids may be considered. Following transplantation, protocol biopsies are needed to detect early recurrence with the intent of complement inhibition.

## Introduction

Membranoproliferative glomerulonephritis (MPGN) is a light microscopic (LM) pattern of injury characterized by mesangial hypercellularity, endocapillary proliferation, and basement membrane reduplication (“double contours”), features that typically result in a lobular accentuation (1). Before considering MPGN as a primary glomerulopathy, secondary causes must be ruled out, most notably infection, autoimmune disease, monoclonal gammopathy, and chronic thrombotic microangiopathy. Some series of primary MPGN exclude monoclonal gammopathies, but others include them.

**Figure 1:**
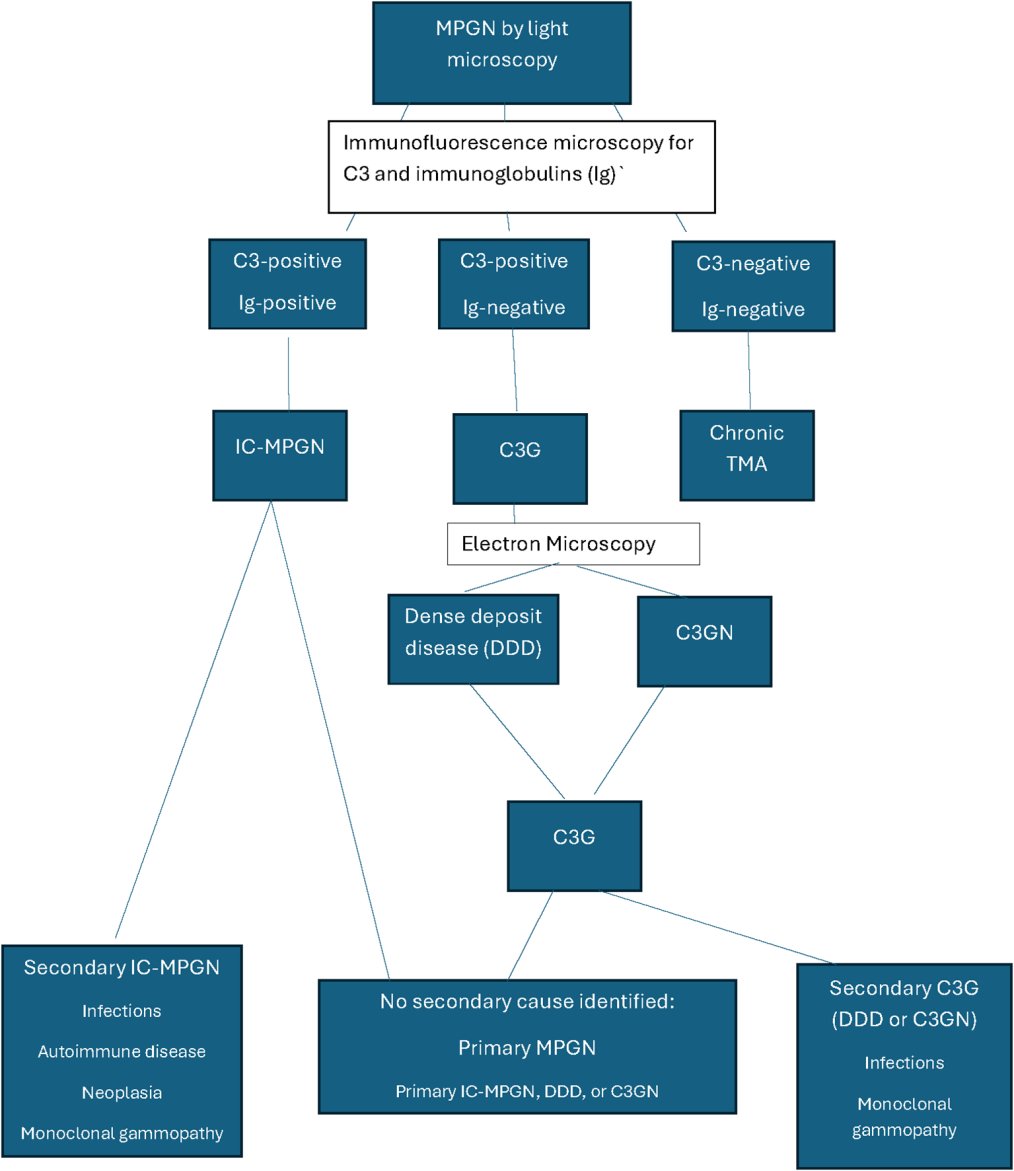
Approach to the light microscopic finding of membranoproliferative glomerulonephritis (MPGN). Immunofluorescence microscopy (IFM) is critical and will differentiate various subtypes. If C3 is negative, the most likely diagnosis is chronic thrombotic microangiopathy which can be confirmed by electron microscopy (EM); further work-up can be directed at potential causes. If C3 is positive, the main differential is between immune-complex mediated MPGN (IC-MPGN), which shows immunoglobulin staining of equal or even greater intensity than C3, and C3 glomerulopathy (C3G), where immunoglobulins are negative or at least 2 orders-of-magnitude less intense than C3. C3G can be further subdivided by EM into dense deposit disease (DDD), characterized by highly osmiophilic, ribbon-like deposits replacing the lamina densa, or C3 glomerulonephritis, characterized by subendothelial +/- subepithelial electron dense deposits. Before considering C3 positive MPGN as primary MPGN, secondary causes must be excluded. For IC-MPGN, these include infections, autoimmune disease, and neoplasia (including monoclonal gammopathy). For C3G (DDD and C3GN) infection- related glomerulopathy and monoclonal gammopathy must be excluded. After such exclusions, IC-MPGN, DDD, and C3GN are considered primary MPGN. Evaluation for genetic mutations and autoantibodies affecting complement regulation should proceed as outlined in the text for all 3 subtypes.

Primary MPGN, the topic of this review, is an ultrarare disease (incidence of 1–3 per million population). Diagnosis requires immunofluorescence microscopy showing significant glomerular basement membrane staining for C3, with or without associated immunoglobulins (**Figure 1**). Primary MPGN is now characterized as either immune-complex mediated (IC-MPGN), when immunoglobulins stain with equal or greater intensity than C3, or as C3 glomerulopathy (C3G) when C3 staining is at least 2 orders of magnitude greater than any immunoglobulins that may or may not be present. C3G is divided by electron microscopic findings (EM) into C3 glomerulonephritis (C3GN), characterized by electron-dense subendothelial deposits with or without subepithelial deposits, and dense deposit disease (DDD), characterized by highly electron-dense, ribbon-like deposits within the glomerular basement membrane. If EM is not available, enrichment of apolipoprotein E, which can be detected by mass spectrometry or immunohistochemistry, indicates DDD is more likely than C3GN (2, 3). Notably, C3G may have a variable LM appearance, including mesangial proliferative, endocapillary proliferative, crescentic, or sclerosing patterns, but such variants are still considered part of the primary MPGN spectrum based on the predominant C3 staining.

## Clinical manifestations

Clinical manifestations of primary MPGN are variable and range from asymptomatic urinary sediment abnormalities with or without reduced GFR to nephrotic syndrome to chronic nephritic syndrome to a rapidly progressive course. Primary MPGN occurs at any age but is more common in children. Although the spectrum of clinical manifestations is similar when comparing children with adults, differences in the frequency and severity of particular syndromes are noted. For example, Nakagawa et al. surveyed the Japan Renal Biopsy Registry from 2007 to 2015 and identified 332 cases of primary MPGN (4). Nephrotic syndrome was significantly more frequent in adults (40.4%), especially the elderly (54%), than in children (14.9%). A chronic nephritic picture (subnephrotic proteinuria with hematuria) was more common in children (66.2%) than adults (34.4%) or the elderly (31.2%). Importantly, DDD was excluded, and it was not specified whether cases were IC-MPGN or C3GN. Other series from Brazil (5) and Russia (6) confirm a predominance of nephrotic range proteinuria, usually with hematuria, in adults. In children, Bajeer et al. confirmed a relatively high incidence (> 60%) of nephritic syndromes (acute or rapidly progressive GN) (7). The long-term outlook of primary MPGN is poor with about 50 - 60% dying or reaching kidney failure in 10 years (8). Overall, adult-onset primary MPGN progresses more often to ESKD than cases with pediatric onset (9, 10). Recent series suggest a similar prognosis comparing IC-MPGN with C3G both in children (7, 11) and adults (10, 12).

## Pathogenesis of MPGN

Central to the pathogenesis of MPGN (herein used as a blanket term to encompass primary IC-MPGN, C3GN, and DDD, including those C3G cases with other histologic patterns) is dysregulation of the alternate pathway of complement (AP) as indicated by obvious C3 glomerular staining. A similar LM appearance is produced by chronic thrombotic microangiopathies (TMA), such as antiphospholipid antibody syndrome and atypical hemolytic uremic syndrome (aHUS), although C3 staining will not be found. Interestingly, a similar AP dysregulation underlies aHUS as in MPGN but involves predominantly tissue-based AP dysregulation, as opposed to MPGN in which AP dysregulation involves predominantly the fluid phase.

## The complement system

**Figure 2:**
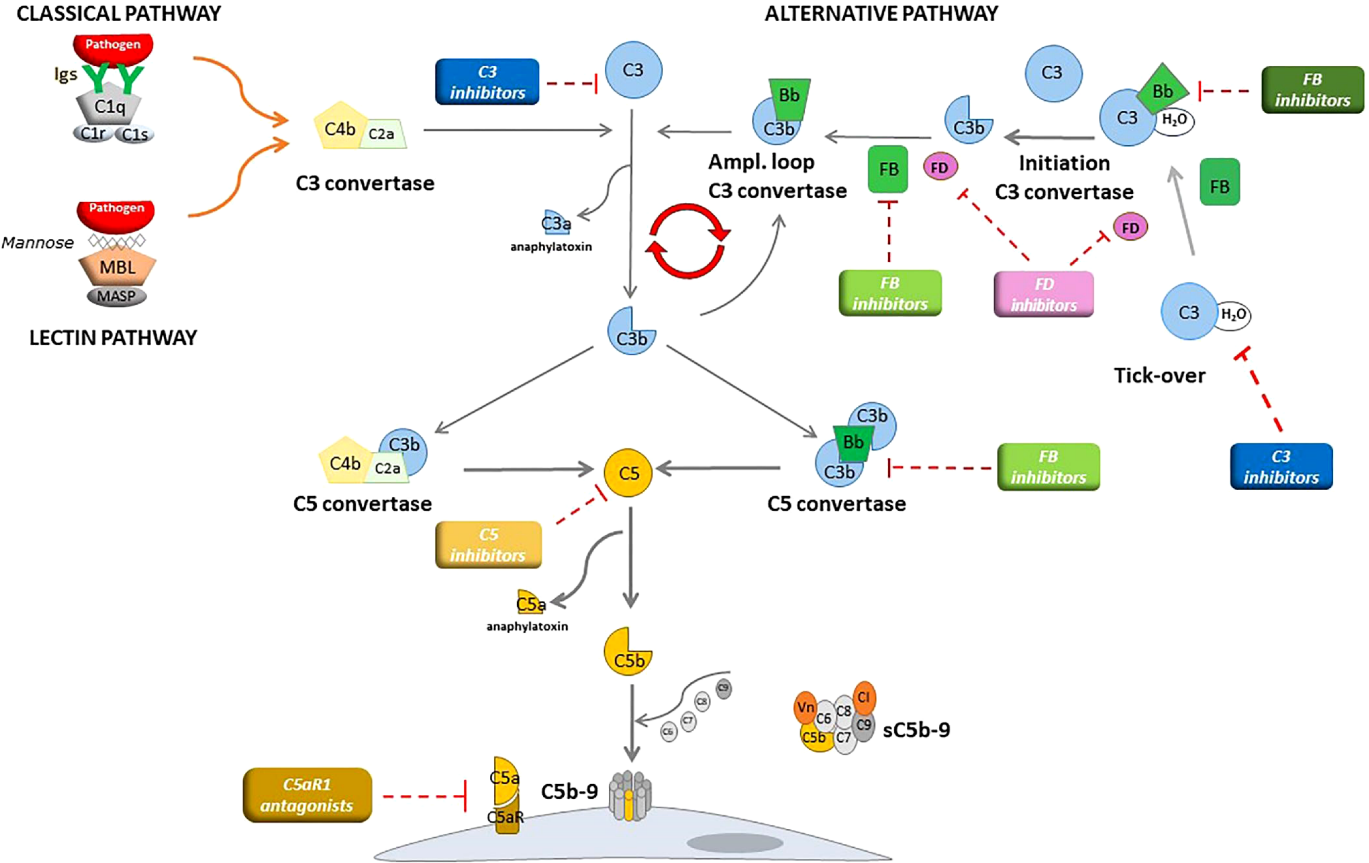
The complement cascade. The classical pathway is activated by the binding of C1q to antibody–antigen complexes, while the lectin pathway is activated by the binding of mannose-binding lectin (MBL) to mannose residues, which activates mannose-binding lectin serine peptidase (MASP) proteins. Either process results in the formation of the classical/lectin, C3 convertase complex that cleaves C3 to C3b and the anaphylatoxin C3a. The alternative pathway is continuously activated in plasma by low-grade hydrolysis of C3 (C3H2O, tick-over) that together with factor B, forms the initiation C3 pro-convertase. FD cleaves factor B to form the active alternative pathway initiation C3 convertase that cleaves C3 to C3b. Complement activation is then amplified by the covalent binding of C3b produced by all the three pathways to hydroxyl groups on cell surface carbohydrates and proteins of target cells, such as bacterial cells. This C3b binds factor B, to form the amplification loop C3 convertase C3bBb. C3b also binds to the C3 convertases, forming the C5 convertase enzymes of the classical/lectin (C4bC2bC3b) and of the alternative (C3b2Bb) pathways that lead to C5 cleavage and the formation of the anaphylatoxin C5a and of the membrane attack complex, composed of C5b, C6, C7, C8 and many copies of C9. The colored rectangles denote the categories of complement inhibitors tested in C3G and Ig-MPGN and the red dashed lines mark their targets. Taken from: Marina Noris, Giuseppe Remuzzi, C3G and Ig-MPGN—treatment standard. *Nephrol Dial Transplant*. 2024; 39(2):202–214(74).

The complement cascade is composed of approximately 30 plasma or membrane-bound proteins (see **Figure 2**) (13, 14). There are 3 initiating pathways for the complement cascade that result in activation of the C3 convertase first, and subsequently the C5 convertase. The classic pathway (CP) involves C1q binding to antigen-antibody immune complexes that results in activation of C4 and then C2 to produce C4b2a, the C3 convertase of the CP. The lectin pathway (LP) is activated by mannose-binding lectins attaching to carbohydrate moieties on microbial cell walls to activate mannose-binding-lectin-associated serine proteases, which serve to activate C4 and then C2 to produce the C3 convertase of the LP, also C4b2a. The AP is constitutively active with spontaneous hydrolysis of C3 to form C3(H2O). Circulating complement Factor D (CFD) then cleaves complement Factor B (CFB) into Ba and Bb, the latter forming the soluble C3 convertase C3(H20)Bb which then cleaves C3 into the anaphylatoxin C3a and C3b. C3b binds to cell surfaces, where it can bind CFB to form C3bB that then is cleaved by CFD to form C3bBb, the main C3 convertase of the AP (14). Properdin stabilizes C3bBb, increasing its half-life 5–10 times. C3bBb can amplify the C3 convertases of all 3 pathways by producing more C3b. The result is activation of the C5 convertases (C4b2aC3b of the CP/LP and C3bBbC3b of the AP, also stabilized by properdin) that cleaves C5 into C5a, a potent anaphylatoxin, and C5b, the latter which initiates the membrane attack complex (MAC) C5b-9 on cell surfaces.

Since the AP is constitutively active, regulation is essential to prevent overactivation and C5 cleavage on host cells. Fluid phase regulators include complement Factor H (CFH), which accelerates C3bBb decay, thereby regulating both fluid phase and cell-based AP activity, and complement Factor I (CFI), which inhibits C3b (with help from CFH). Membrane-based regulators include CFH, membrane cofactor protein (MCP, CD46), decay accelerating factor (DAF), CD59, and complement receptor 1 (CR1, CD35) (13).

## Mutations and MPGN

Overall, about 20% of MPGN cases are caused by classic Mendelian type mutations with variable penetrance and expressivity (13) (see **Table 1**). The genes with the strongest association with MPGN include C3 (*C3*, all genes in italics) and CFB (*CFB*), the components of the AP C3 convertase, as well as its regulators, CFH (*CFH*) and CFI (*CFI*). Mutations in complement Factor H-related proteins 1-5 (*CFHR1-5*) are also a well-known cause of MPGN. CFHRs 1- to 5 are structurally related to CFH and antagonize its ability to regulate complement. Mutations include deletions, duplications with copy number variations, and hybrid gene formations (15–17). Mutations in *MCP* have been associated with MPGN, but the link with aHUS is much stronger (18). Mutations in diacylglycerol kinase epsilon (*DGKE*), not a complement system protein but rather involved in intracellular lipid signaling, are a rare cause of MPGN (19). Mutations in the coagulation proteins thrombomodulin (*THBD*) and plasminogen (*PLG*) have been linked to aHUS but not MPGN. Mutations in properdin (*CFP*) have not been found to cause MPGN, although the protein may interact with mutations in the C3 convertase.

**Table 1:** Genetic analysis of primary MPGN.

The following complement system-related genes (in italics) should be assessed				
Protein (gene)	Function	Mutation frequency	Expected phenotype	Associated serum biomarkers
C3 (*C3*)	Complement protein, substrate for C3 Convertase	5 – 10%	IC-MGN or C3G (no preference)	C3 low and C5b-9 elevated but frequency uncertain; C4 normal
Complement Factor B (*CFB*)	Essential protease that forms C3 convertase together with C3b	Rare (< 5%)	More often C3G than IC-MPGN	Low C3 common, C5b-9 elevated in some; C4 normal; elevated Ba/Bb fragments
Complement Factor H (*CFH*)	Main negative regulatory protein of AP amplification; both fluid phase and membrane bound	5 – 20%	IC-MGN or C3G (no preference)	Low C3 is hallmark; high C5b-9 is typical; C4 normal; Factor H may be low
Complement Factor I (*CFI*)	Serine protease that inactivates C3b; requires cofactors (factor H, CD35, or MCP (CD46)	Rare (< 5%)	IC-MGN or C3G (no preference)	Both low C3 and high C5b-9 are typical; C4 normal; Factor I may be low
Membrane Cofactor Protein (*MCP*)	Membrane protein regulator of complement; a cofactor for Factor I	Rare		C3 low; C5b-9 variably high; C4 normal
Complement Factor H-Related protein gene family 1 – 5 (*CFHR1-5*)	CFHR1–5 are tandem CFH−like proteins that compete with Factor H hence reducing regulation	Deletions, duplications of intragenic segments, extra gene segments, or large genomic segments* < 5%	C3G much more likely than IC-MPGN	Low C3; variable levels of CFHRPs; C4 normal Factor H antibodies with homozygous CFHR1-CFHR3 deletion

*The Factor H-CFHR gene cluster is on chromosome 12: the 5 *CFHR1–5* genes are located downstream from the *CFH* gene in the order: *FH, CFHR3, CFHR1, CFHR4, CFHR2, CFHR5.* C3G may be caused by heterozygous *CFHR* gene mutations with intact *Factor H*, or homozygous/compound heterozygous *Factor* H gene mutations with intact CFHR genes. Examples: the *CFHR3::CFHR1* deletion results in a CFHR3/CFHR1 hybrid with abnormal regulatory properties. Cypriot C3 glomerulopathy (CFHR5 nephropathy) is caused by a heterozygous internal duplication within the CFHR5 gene producing an abnormal CFHR5 protein. Other examples include whole−gene *CFH* deletion and *CFHR4* duplication. Other hybrid genes have been described. For a detailed discussion see (15, 102–104)

CNV, copy number variations; C3G, C3 glomerulopathy; IC-MPGN, immune complex membranoproliferative glomerulonephritis.

It is of interest to compare the loci with mutations common to both aHUS and MPGN. Over 250 pathologic *CFH* mutations have been described causing aHUS and over 50 for MPGN (13). The mutations responsible for aHUS cluster in the C-terminal region, and those causing MPGN in the N-terminal region; however, there is much overlap. Although a given mutation is usually associated with either aHUS or MPGN, some mutations may cause either phenotype. This indicates other factors determining the phenotype of a given patient (aHUS or MPGN), including other genetic modifiers, coexisting autoimmune phenomena, or environmental influences. A similar phenomenon occurs with mutations in the other genes common to both disorders (*C3, CFB, CFI, MCP*).

Several studies assessed the underlying genetic make-up of MPGN. Some combination of 6 genes is typically evaluated (*C3, CFB, CFH, CFI, MCP, CFP*), as well as deletions and gene rearrangements of the *CFHR1–5* region. Other non-complement system genes implicated in aHUS, but not yet in MPGN, include coagulation system genes (*THBD* and *PLG*) and the intracellular signaling molecule diacylglycerol kinase epsilon (*DGKE*). In an earlier study, Servais et al. analyzed a cohort of 134 French patients (49 IC-MPGN, 56 C3GN, 29 DDD) for mutations in the *CFH*, *CFI*, and *MCP* genes and found mutations in 24 (17.9%), including 17 *CFH*, 6 *CFI*, and 1 *MCP*. Approximately half also had autoantibodies targeting and stabilizing the AP C3 convertase, termed C3-nephritic factors (C3NeF, *vide infra*), as well as did 53 other patients without mutations (20).

Iatropoulos et al. performed next-generation sequencing (NGS) to evaluate *CFH, CD46, CFI, CFB, C3* and *THBD* in 140 Italian patients (67 IC-MPGN, 21 DDD, and 52 C3GN). They found likely pathogenic variants in 24/135 unrelated patients (18%) with no differences between the 3 groups. The patients were significantly enriched with likely pathogenic variants in all 6 genes as compared to controls from the 1000 Genome Project (21). C3 mutations were most common, found only in IG-MPGN and C3GN but not in DDD, whereas mutations in complement regulatory genes were found in all 3 subgroups. Importantly, the risk was mainly increased when the mutations were combined with common susceptibility variants potentially explaining incomplete penetrance. C3 nephritic factors (C3NeFs, *vide infra*) were found in 57/116 (49%) tested patients, especially with DDD (78%). Kidney survival was not different in the 3 histologic subgroups, but it was worse in those lacking both mutations and C3NeFs.

Coupling NGS with targeted genomic enrichment, Bu et al. evaluated the Genetic Complement-Mediated Renal Disease Panela panel of 10 genes (*CFH, CFI, CFB, C3, CFHR5, CD46, DKGE, ADAMTS13, THBD, PLG*, plus copy number variation in *CFHR3-CFHR1*) in 193 patients, including 37 C3G and 147 TMA. They found a positive genetic diagnosis (including pathogenic/likely pathogenic/variants of undetermined significance) in 43% of C3G patients (22). Whereas C3G patients had variants in both C3 convertase (*C3* and *CFB*) and AP regulator genes, variants in aHUS patients clustered only in AP regulator genes.

Mueleman et al. evaluated 398 French patients (102 IC-MPGN, 296 C3G) for rare variants (MAF < 0.1%) of the CFH, CFI, and C3 genes and found 53 rare variants, including 30 in *CFH*, 13 in *CFI*, and 10 in *C3*, in 66/398 patients (17%, including 55 C3G and 11 IC-MPGN) (23). Thirty-eight of the 53 variants (72%) were classified as pathogenic, including 20 of 30 *CFH* variants and 11 of 13 *CFI* variants. A total of 48 patients had a pathogenic variant. Compared to controls from the 1000 genome project, the C3G group harbored a higher frequency of pathogenic variants in all 3 genes, whereas the IC-MPGN group only in *CFH* and *CFI* genes, but not *C3*. Stigmata of TMA were significantly more frequent with *CFI* variants (5/14) as compared to *CFH* and *C3*. Kidney survival was significantly worse in patients harboring variants than in those that did not, unlike the Italian series of Iatropoulos et al. (21) noted above.

By contrast, Levine et al. performed whole genome sequencing in 146 primary MPGN patients (46.5% IC-MPGN, 34.2% C3GN, 19.3% DDD) and 6,442 controls and found no significant enrichment of rare variants in complement pathway genes or other genes associated with aHUS (24). However, they did find a significant association with an HLA class II haplotype that was replicated in a separate group, a result that suggests primary MPGN is an autoimmune disease.

A Kidney Disease Improving Global Outcomes (KDIGO) controversies conference on the role of complement in kidney disease suggests that all patients with primary IC-MPGN and paraprotein-negative C3G should have genetic analysis given that rare variants (MAF < 0.1%) are found in about 20%, especially involving *C3, CFB, CFH, and CFI* (25). Such rare variants are associated with reduced serum concentrations of their corresponding proteins (21–23). Additionally, non-monogenic and common genetic risk factors for MPGN may be identified that of themselves are insufficient to cause disease, but they may alter the expressivity of pathogenic rare variants if also present (26, 27). In our opinion genetic testing should be performed for primary MPGN (see **Table 1**).

## Autoantibodies in MPGN

In addition to mutations, autoantibodies against various complement components are frequently found in MPGN (see **Table 2**). The most common autoantibodies are nephritic factors which target the C3 and/or C5 convertases. C3 nephritic factors (C3NeF) target and stabilize the AP C3 convertase (C3bBb) and are found in 70 – 80% of DDD cases, 40 – 50% of C3GN cases, and 40 – 50% of IC-MPGN cases (28). Serum C3 levels are low in most C3NeF-positive cases, but a minority may have persistently normal C3 levels (29). C4 nephritic factors (C4NeF) stabilize the C3 or C5 convertases of the CP and LP (C4b2a and C4b2aC3b) and are found in <10% of MPGN cases. Serum C4 levels are typically normal, and C4NeFs can coexist with C3NeFs (30). C5 nephritic factors (C5NeF) target the AP C5 convertase (C3bBbC3b) and are found in about 30% of DDD cases and in about 70% of C3GN cases and commonly coexist with C3NeFs (31). C5NeFs correlate with both low C3 serum levels and elevated serum C5b-9 levels (31).

**Table 2:** Autoantibodies and serum complement levels to consider measuring in primary MPGN.

Antibodies, Assays, and Components	Frequencies
Antibodies	C3NeFs (40 – 80%) C4NeFs (10 – 15%) C5NeFs (~50%) Anti-Factor H antibodies (3 – 6%) noted with homozygous CFHR1-CFHR5 deletions Anti-Factor I antibodies (extremely low prevalence) Anti-C3 antibodies (5 – 15%)* Anti-Factor B antibodies (6 – 11%)*
AP functional assays**	Frequent
Complement components	C3 (low in 40 – 80%) C4 (low in 10 – 20%) C5b-9 (elevated in 50 – 80%) Complement Factor H (< 10%)***

*distinct from C3NeFs which interact with C3 convertase as a whole (34).

**Various assays to assess functional activity of the alternate pathway are available. See (105). Also, assumes secondary causes already excluded. Requires proper handling and may require a specialized laboratory.

***associated with mutations in Factor H gene or autoantibodies.

NeF, nephritic factor.

CFH autoantibodies are found in about 3% of MPGN cases and in about 10% of aHUS cases where they are associated with deletion of *CFHR3-CFHR1* (over 90% with anti-CFH antibodies have this deletion) (32). Rarely, a monoclonal protein may have anti-Factor H activity and produce MPGN, a form of monoclonal gammopathy of renal significance (33). The epitope specificity of CFH autoantibodies may determine the phenotype. In MPGN, the antibodies tend to target the N-terminus, thereby impairing fluid phase complement regulation. By contrast, in aHUS these antibodies tend to target the C-terminus to impair control on the cell surface, mimicking the clustering of mutations with associated phenotypes (32). Autoantibodies against CFB and/or C3, the constituents of the AP C3-convertase, may be found in up to 10% of MPGN cases (34).

The KDIGO controversies conference recommends screening for C3/5NeFs in addition to complement biomarker profiling (C3, C4, C5b-9, etc.) as NeFs may be found in 40 – 80% of cases and indicate an autoimmune pathogenesis (25). As outlined above, a majority of MPGN patients have either complement gene mutations or autoantibodies, with many having both. However, a significant minority have neither, and such “double-negative” patients, perhaps one-quarter of MPGN patients, may have the worst prognosis (21, 30).

## MPGN recurrence following transplantation

MPGN recurs following kidney transplantation. At least 7 case series together transplanting 318 total patients with IC-MPFN or C3G as their primary disease found a total of 123 recurrences (38.7%) (35–41). The largest series from The Spanish Group for the Study of Glomerular Diseases (GLOSEN) included 132 patients with IC-MPGN and 34 with C3G; recurrences occurred in 20/132 (15%) with IC-MPGN at a median of 30 months and in 21/34 (61.7%) with C3G at a median of 14 months (39). Graft loss occurred in 13 of 20 recurrent IC-MPGN cases and 12/21 recurrent C3G cases. In other studies, the longest reported time to recurrence was a median of 1.2 years in the 7 patients that recurred out of 41 transplanted Swiss patients (41). By contrast and aided by routine protocol biopsies, Tarragon et al. found C3G recurrence in 16 of 18 patients (11/12 with C3GN and 5/6 with DDD) at a median of 33 days (40). Initial detection of recurrence was predominantly established by immunofluorescence microscopy and electron microscopy as light microscopic changes were very subtle. Clinical manifestations were mild (only 31% had UPCR > 300 mg/g). Importantly, repeat protocol biopsies at 1 and 2 years revealed persistent C3 staining, activity increasing at 1-year that persisted to 2 years, and chronicity increasing from years 1 to 2.

There is no reliable way to predict recurrence of MPGN following transplantation. A workgroup established by the American Society of Transplantation Kidney Pancreas Community of Practice and the Pediatric Community of Practice recently concluded that genetic variants do not define recurrence risk nor susceptibility to specific medications and should not guide decisions regarding transplant candidacy; however, some members recommended consideration of genetic screening (*C3, CFB, CFH, CFI*, and *CFHR1-5*) and evaluation for autoantibodies (42). Likewise, the KDIGO controversies conference suggests recurrence risk is unreliably determined by genetic testing given variable penetrance and phenotypic dissimilarities (25). Pretransplant testing typically includes measuring serum levels of complement proteins (CFB, C3, C4, C5b-9), regulators (CFH, CFI, MAC), and autoantibodies (C3/5NeFs, anti-CFH, anti-CFB). Attieh et al. opined that demonstration of autoantibodies or abnormal measurements of complement protein levels should not influence transplantation suitability (43).

## Prognosis in MPGN

Given the variability in clinical presentation, clinical course, pathology, and pathophysiology, attempts have been made to better predict long-term outcome, as well as to hopefully guide precision-based therapy for the individual patient. Earlier studies indicated worse kidney survival with DDD versus C3GN (44), at least in adults (20). More recent data, however, indicate no significant difference in risk of kidney failure (KF) between C3GN, DDD (45), or IC-MPGN (10, 46, 47).

Bomback et al. studied 111 C3G patients at Columbia University (24 with DDD, 87 C3GN) and developed a C3G-histologic index comprised of 7 active components and 4 chronic components. There was no difference between DDD and C3GN in the hard endpoint of doubling of serum creatinine and/or progression to KF. In multivariable analyses, eGFR at diagnosis was the only significant clinical predictor of the adverse kidney outcome or death. In additional analyses that included pathology, interstitial fibrosis and tubular atrophy were significant predictors, as were total activity and chronicity scores in a third analysis. Baseline proteinuria was found to be a significant risk for adverse renal outcomes in some studies (48–51), but not in all (47). Perhaps more important is change in proteinuria over time. In a study from GLOSEN, Caravaca-Fontan et al. evaluated 85 patients (70 C3GN and 15 DDD) for the risk of KF based on the longitudinal change in proteinuria (52). Over a median of 42 months, 25% reached the endpoint. An inverse relationship between the change in proteinuria and the slope of eGFR was found. Considering the change in proteinuria continuously, a doubling resulted in a 2.5-fold increase in KF. A ≥ 50% reduction during follow-up resulted in reduced KF by 21% (p<0.001). Furthermore, ≥ 50% reductions at either 6- or 12-months were significantly associated with reduced KF (by 4% at 6-months, p=0.02 and by 17% at 12 months, p<0.001).

In a multinational study, Ghaddar et al. followed 225 European patients (157 C3GN, 37 DDD, and 31 IC-MPGN) over a median of 3.7 years for a combined endpoint of ≥ 30% decline in eGFR or KF. In a multivariable model, lower baseline eGFR, a paraprotein (present in 5%), and interstitial fibrosis were significantly associated with higher risk. In contrast, white ethnicity, lower C4 levels, and native kidney disease (versus recurrence in a transplant) were associated with lower risk. Baseline proteinuria was not associated with increased risk, but in patients with baseline proteinuria > 1 g/day, a ≥ 50% reduction to < 1 g/day significantly reduced the risk by 65%. Modeling log-transformed proteinuria over time versus the primary endpoint, the risk significantly increased at 1 g/day.

In the largest study to date, the UK National Registry of Rare Kidney Diseases (RaDaR) analyzed data from 371 incident patients (138 with C3GN, 65 with DDD, and 168 with IC-MPGN) followed for a median of 11 years for development of KF that occurred overall in 138 patients (40%) (10). There was no difference in KF rate between C3G (42%) and IC-MPGN (37%). Data at diagnosis and 1 year from 91 patients (44 with C3G and 47 with IC-MPGN) allowed assessment of how relatively short-term changes in eGFR and proteinuria affected development of KF. Overall, baseline proteinuria was not associated with KF by multivariable analysis, whereas female sex and lower CKD stage at diagnosis were. In the 91 with evaluable data, annualized 2-year eGFR slope was significantly associated with KF, but only modestly (by 68% - 99%) for a 10 ml/min/1.73m^2^ decline. More impressively, absolute reductions of urine protein in the combined cohort (91 patients) between 0 and 12 months greatly reduced risk: attaining < 100 mg/mole creatinine (< 880 mg/g) reduced the risk by 90% (p<0.0001). Furthermore, a 50% decline from baseline at 6 or 12 months significantly reduced risk (by 38% - 60%), and a 50 mg/mmole decline significantly reduced KF at 1 year (37%). There was no substantial difference between the 44 with C3G and the 47 with IC-MPGN.

Overall, the data from these 3 studies (GLOSEN, Ghaddar et al. and RaDaR) indicate that proteinuria at 1 year is at least a “reasonably likely” surrogate endpoint, if not a “validated” one that can be used as an endpoint of randomized controlled trials (RCTs) of therapy.

Chauvet et al. analyzed a French national cohort of 165 patients (64 children, 101 adults) with C3G (no IC-MPGN) and emphasized the role of baseline serum complement abnormalities on progression to KF with a median follow-up censored for KF of 47 months (53). Patient were divided into 4 groups based on C3 (low in 59%) and C5b-9 levels (high in 51%): normal C3/normal C5b-9 (47 patients, 30 adults), low C3/high C5b-9 (53 patients, 37 adults), low C3/normal C5b-9 (34 patients, 20 adults), and normal C3/high C5b-9 (21 patients, 14 adults), the latter 2 groups suggesting disparate overactivity between the C3 and C5 convertases, respectively. By multivariable analysis in the whole cohort, adult onset, reduced eGFR, disease-predicting genetic variants, and low C3/normal C5b-9 as well as normal C3/high C5b-9 predicted risk for KF. However, analyzing by age, the only significant factor in children was presence of a mutation, whereas in adults, disease-predicting mutations, low eGFR (< 60), and either low C3/normal C5b-9 or C3/high C5b-9 remained independently significant. In adults, assigning a score of 1 for each of the 3 factors significantly determined median survival free of KF: score= 0, 20 patients (median undefined), score 1, 28 patients (median 115 months), score 2, 23 patients (median 21 months), and score 3, 4 patients (median 3 months).

Iatropoulos et al. studied 173 patients from an Italian registry, including 88 with C3GN, 25 with DDD, and 80 with IC-MPGN. By performing unsupervised hierarchical clustering utilizing histologic, clinical, immunologic, and genetic parameters, they identified 4 distinct clusters having somewhat different pathogenetic patterns (54). Cluster 1 (62 patients, including 42 C3GN, 4 DDD, and 16 IC-MPGN) had a high prevalence of genetic mutations and/or C3NeF, low serum C3, and very high C5b-9 levels with a young age of onset (15 years). Cluster 2 was similar except for strong immunoglobulin staining (32 patients, including 2 C3GN and 30 IC-MPGN). Cluster 3 (33 patients, 4 C3GN, 21 DDD, 8 IC-MPGN) was similar to cluster 1 except for normal or mildly elevated C5b-9, more crescents, and more intra-membranous deposits. Cluster 4 had lower mutations and antibodies, later age of onset, and more sclerosis. Cluster 3 had a significantly higher risk of KF.

Garam et al. performed a validation cluster analysis on 92 eastern European patients (37 C3GN, 11 DDD, 44 IC-MPGN) and also found 4 clusters with features overlapping the clusters of Iatropoulos et al (55). Significant differences in progression to KF were found. Patients in clusters 3 and 4 had more progression to KF than those in clusters 1 and 2.

Expanding on their Italian registry (54) to include 295 patients (125 with C3GN, 35 with DDD, and 135 with IC-MPGN), Benigni et al. applied cluster analysis utilizing 29 clinical, histological, genetic, and biochemical variables to define now 5 clusters by separating cluster 4 of the original analysis into 2 clusters with an eye on personalizing therapy (56). Cluster 1 (70 patients) had low C3, high C5b-9, mesangial and subepithelial deposits with a mean age of onset of 16 years, enriched with C3GN cases (69%). Nephritic factors were present in one-third, predominantly C5NeF that explains the high serum C5b-9. Factor B antibodies were more common possibly driving AP activation. Presumably, these cases would benefit most from Factor B inhibition (e.g., iptacopan, *vide infra*) or Factor 5aR blockade to reduce inflammation (e.g., avacopan, *vide infra*). Cluster 2 (67 patients) was similar (low C3, high C5b-9, NeF positivity) but had positive immunoglobulin staining that indicates a contribution of classical pathway activation (perhaps as an initiating factor). Hence the suggestion that these cases may benefit from C3 inhibition, since both classic and alternate pathways may be involved (e.g., pegcetacoplan, *vide infra*). Cluster 3 (41 patients) had low C3 but minimally elevated C5b-9 (suggesting Factor B inhibition preferred as opposed to C5 inhibition) with intramembranous highly electron-dense deposits; 25 of 35 DDD cases were in this cluster. Cluster 4 (44 patients) had essentially normal serum complement levels, more glomerulosclerosis and interstitial fibrosis, and an older age of onset (35 years). Cluster 5 (73 patients) also had essentially normal serum complement but less sclerosis than cluster 4 and an earlier age of onset (19 years). For clusters 4 and 5, agents targeting cell-surface complement activation (e.g., eculizumab, *vide infra*) may be optimal.

The 5 clusters had distinct kidney outcomes over approximately 10 years. The best kidney survival was cluster 1 (87%), the worst was cluster 4 (48%). Renal survival was 77% in cluster 2, 83% in cluster 3, and 69% in cluster 5. The hazard ratio for KF for cluster 4 versus cluster 1 was 7.82 (p=0.0012). At the end, 74% of cluster 1 had normal kidney function, and 44% had normal-range proteinuria compared to 0% of cluster 4. Standard histopathological differentiation (C3GN, DDD, IC-MPGN) did not predict outcomes. A publicly available web application allows assignment of a given patient to a specific cluster based on data available at the time of diagnosis: https://www.izbi.uni-leipzig.de/lha/interactive-mpgn-classification-tool/. This would be most useful if differential responses to various complement inhibitors suggested above can be demonstrated in randomized controlled trials (RCTs). Otherwise, considerations for immunosuppression should be based on conventional biomarkers, such as proteinuria (baseline and over time), eGFR, and biopsy findings (activity/chronicity).

## Treatment

The optimal treatment for MPGN remains uncertain. It is an ultrarare disease that evolves over years with a variable clinical course. Most likely, one size does not fit all, and therapy should be individualized as much as possible. Generalized supportive care should be implemented, as for all proteinuric glomerular diseases, including smoking cessation, weight loss (if overweight or obese), salt restriction, strict BP control (< 120/80), renin-angiotensin-aldosterone system inhibition, and SGLT2 inhibition (57).

Personalized therapy for otherwise primary IC-MPGN and C3G begins with exclusion of a paraprotein. Proliferative glomerulonephritis with monoclonal immunoglobulin deposits, a form of MGRS, frequently presents with membranoproliferative histology, although other patterns are also possible, such as mesangial proliferative glomerulonephritis, endocapillary proliferative glomerulonephritis, and rarely an atypical membranous pattern (58). Large series of IC-MPGN find a paraprotein in approximately 20% (46) – 40% (59) of otherwise idiopathic cases, with a similar frequency in C3G. Immunofluorescence microscopy findings of kidney biopsies in IC-MPGN typically mirror the associated paraprotein with monoclonality serving as a clue to the etiology. In the case of C3G, monoclonal deposits are not found in the kidney biopsy. Paraproteins presumably cause the disease by interfering with alternate complement pathway regulation analogous to C3 and C5 nephritic factors or possibly anti-CFH antibodies.

The benefit of paraprotein clone-directed therapy in C3G was nicely demonstrated a series of 50 paraprotein-positive patients out of 201 total French C3G patients treated with clone-directed chemotherapy (29 patients), immunosuppression (8 patients), or conservative therapy (13 patients) (60). Chemotherapy was clone-directed, and included alkylating agents-dexamethasone in 5, bortezomib-dexamethasone-based regimens in 22, and prednisone-rituximab in 2 (CLL patients). Hematological response rate occurred in 17/29 chemotherapy patients and 1/20 others. Of the 17 with hematologic response, 15 (83%) had a renal response (partial or complete), as compared to 5 of 18 (28%) without a hematologic response. Comparing patients over the age of 50 with C3G, those with paraprotein-associated disease had similar overall patient survival but significantly worse kidney survival in comparison to similarly aged C3G patients without a paraprotein.

## MMF +/- corticosteroids

Earlier uncontrolled case series suggested a benefit to MMF and steroids (61, 62). The GLOSEN group retrospectively studied 60 patients with C3GN (no DDD) followed for a median of 47 months and treated either with MMF and steroids (22 patients), steroids +/- cyclophosphamide (18 patients), or no immunosuppression. No patients treated with MMF/steroids had doubling of serum creatinine or reached ESKD versus 7/18 and 3/18, respectively, in the steroid +/- cyclophosphamide and no immunosuppression groups. Remission was significantly higher (86%) in MMF-treated patients compared to the other 2 groups (50 and 25%) (63).

The GLOSEN group expanded their retrospective analysis to include 97 patients (81 C3GN, 16 DDD) treated with MMF/steroids (42 patients), other immunosuppressants (29 patients), eculizumab (9 patients), or conservatively (17 patients). They analyzed remission rates and progression to KF with a median follow-up of 46 months (51). Respective remission rates were 79%, 24%, 33%, and 18%, and progression to kidney failure occurred in 14%, 59%, 67%, and 65%, respectively, results that suggest superiority of MMF/steroids. Restricting analysis to 34 MMF-treated patients propensity matched to 34 patients treated with other immunosuppressants confirmed the reduced progression to kidney failure with MMF. In the overall cohort, remission occurred regardless of underlying complement gene mutations (18 patients) or autoantibody detection (29 patients), although only partial remissions (6/18 patients) occurred with underlying mutations, whereas both complete (10/18 patients) and partial (6/18 patients) remissions occurred in those with autoantibodies.

Avasare et al. retrospectively analyzed 30 C3G cases (29 C3GN, 1 DDD) at Columbia University treated for at least 3 months with MMF/steroids. They found that 20 (67%) responded (10 partial, 10 complete remissions) and 3 progressed to KF (all non-responders). Response rates to other regimens were less robust: 39% of 23 with steroids alone, 29% in 7 with steroids/calcineurin inhibitors, 33% in 6 with steroids/cyclophosphamide, and 29% in 7 with steroids/rituximab. Bharati et al. retrospectively evaluated 17 Indian C3G patients given MMF and found complete remission in 4 and partial remission in 7 for a 54% overall response (64).

Others found no benefit to MMF-based regimens (65–67). Ravindran et al. reviewed the Mayo clinic experience of 114 C3G patients evaluated over a 10-year period (49). Of 24 patients given an MMF-based regimen, there was only 1 complete remission and 2 partial remissions as compared to 13 of 34 treated conservatively (6 complete remissions, 7 partial). Similarly, Caliskan et al. retrospectively compared 27 C3G patients treated with MMF-based regimens to 16 only receiving conservative care and found no significant difference in KF or ≥ 50% decline in eGFR between the groups (48).

Other immunosuppressive therapies for MPGN have been tried in case reports or case series, but data are insufficient to make recommendations. These include cyclophosphamide/steroids (children) (7), rituximab (68), and rituximab combined with belimumab (69).

The 2021 KDIGO guidelines suggest using MMF as first line therapy for patients with C3G (after excluding monoclonal gammopathy), if they are at risk for progression based on moderate-to-severe disease (proteinuria >1 g/d and/or declining kidney function over several months) (57). For IC-MPGN, recommendations were even less specific, including consideration of a limited course of corticosteroids for nephrotic syndrome with preserved GFR or consideration of steroids and immunosuppression if GFR is declining or if there is a rapidly progressive course; if GFR is < 30, they recommend supportive care only (57). We expect these recommendations to be updated given the data on anti-complement therapy.

## Complement inhibition

Central to pathogenesis of MPGN is dysregulated AP activation, and numerous anti-complement agents have been and are being tested (see **Table 3**). The Kidney Health Initiative (KHI), a partnership between the FDA and American Society of Nephrology, convened a Work Group to review available evidence and make recommendations on potentially valid surrogate endpoints for trials of anti-complement therapy (70). Optimally, three specific endpoints should be met: reduction of proteinuria, stabilization or improvement of eGFR, and histologic improvement on repeat biopsy (reduction of C3 staining intensity and reduced activity on the C3G-histology index), although meeting the first 2 may be considered effective. Unfortunately, minimum thresholds were not defined. The results of the following anticomplement therapies should be considered in this light.

**Table 3:** Randomized controlled trials of complement inhibition versus placebo or SOC*.

Medication/target	Trial	Population	Number	Primary endpoint	Result	Comments
Pegcetacoplan C3	NOBLE Bomback (81)	Transplant recurrent C3G or IC-MPGN	13	Reduction of C3 staining on repeat biopsy at week 12 vs SOC only	5/10 pegcetacoplan ≥ 2 OOM reduction; 8/10 ≥ 1 OOM reduction versus 1/3 SOC	Reduced C3G activity score 50% reduction UPCR eGFR stable C3 increased, C5b-9 decreased
	VALIANT Nester (83)	Native or recurrent C3G, native IC-MPGN	124	Log transformed UPCR reduction at week 26 vs placebo	68% vs placebo (p<0.0001)	GFR stabilized and C3 staining reduced.
Danicopan Factor D	Study 204 Nester (84)	C3G	13	Change in histology at 6 months ≥ 30% reduction in 24 urine protein (g/day)	No change in either group histologically Proteinuria reduction: 0/6 given danicopan vs 1/7 placebo	Terminated early due to lack of efficacy Incomplete AP suppression; not sustained during 8-hour dosing period
Iptacopan Factor B	APPEAR-C3G Kavanagh (87)	C3G	74	Log transformed 24-hour UPCR reduction at 6 months vs placebo	35.1% vs placebo (p=0.0014)	30% of iptacopan patients vs 6% placebo reached composite: ≥ 50% reduction UPCR with stable GFR
Avacopan C5a receptor	ACCOLADE Bomback (79)	C3G	57	Percent change from baseline in Histologic activity Index at 26 weeks	Difference 0.0	No difference in chronicity index, UPCR, or GFR Signal suggesting reduced progression of fibrosis in placebo patients given active drug from weeks 26 to 52

*only randomized controlled trials included. For uncontrolled studies, see descriptions in the text.

AP, alternate pathway; C3G, C3 glomerulopathy, includes C3 glomerulonephritis and dense deposit disease; IC-MPGN, immune complex membranoproliferative glomerulonephritis; OOM, orders of magnitude; SOC, standard of care; UPCR: urine protein/creatinine ratio

C5 activation contributes to the pathogenesis of C3G as shown by elevated C5b-9 levels in many patients. The C5 inhibitor eculizumab has been used with earlier case reports indicating benefit (71–73). Numerous other case reports have been published (referenced in (74)). Results in reported case series, however, have been variable.

Bomback et al. treated 6 adult patients C3G (3 native kidney, 3 recurrent in allografts) manifesting as elevated UPCR or AKI with eculizumab for 12 months (75). Two patients had reduced serum creatinine, one partial remission of nephrotic syndrome, and one had histologic improvement despite stable labs. The remaining two had declining kidney function on treatment.

Le Quintrec et al. gave eculizumab predominantly as second-line therapy to 26 patients (13 adults) from France and Quebec and found that 6 had a global response (reduced creatinine and/or proteinuria) and 6 had a partial response; 14 (54%) had no response. Global response was predicted by lower eGFR at initiation, a rapidly progressive course, and greater extra-capillary proliferation (76). On repeat biopsies, C3 staining was not reduced as expected, since eculizumab inhibits complement distal to the C3 convertase.

Ruggenenti et al. published a multicenter prospective off-on-off-on open label trial in 10 Italian patients with elevated C5b-9 levels and urine protein > 3.5 g/day (6 IC-MPGN, 4 C3GN) given eculizumab for 48 weeks followed by a 12-week washout and then another 48 weeks of active therapy (77). The primary outcome was the change in urine protein at 24 and 48 weeks. Proteinuria decreased significantly at both times, rebounded during washout, but did not decrease thereafter. Partial remission was only achieved by 3 patients. All patients had normalized C5–9 during active therapy only, but C3 remained low during active therapy.

Factor H deficient mice develop a lesion analogous to C3GN, which can be significantly ameliorated with concurrent C5 deficiency (78). When factor H deficient mice were exposed to an anti-GBM antibody, excessive neutrophil accumulation was found. Concurrent C5 deficiency abrogated this response, but C6 deficiency did not, thereby indicating a more prominent role for C5a versus C5b-9. A multicenter, double-blind, placebo-controlled phase 2 trial (ACCOLADE) compared the oral C5aR blocker avacopan 30 mg bid to placebo in 57 patients for 6 months, with an open label 6-month phase where all patients received active drug (79). This 12-month format (6 months with placebo control and 6 months open label active drug given to all patients) is the blueprint for such complement inhibition trials for MPGN as recommended by the KHI workgroup (70). The primary endpoint in ACCOLADE was change in the C3G-histology index activity score at the end of 6 months on repeat biopsies. There was no significant difference between the groups in the primary endpoint or in secondary endpoints (chronicity index, urine protein-creatinine ratio (UPCR), or eGFR). However, comparing biopsy changes from baseline to 6 months in the placebo group to subsequent changes from the 6-month to the 12-month biopsies when placebo patients received active drug demonstrated both a reduced percent change and reduced actual change in the C3G-chronicity index, a signal of potential benefit.

A compstatin derivative, pegcetacoplan, binds to both C3 and C3b to prevent further cleavage of C3. The result is inhibition of the alternate pathway amplification loop. By inhibiting the breakdown of C3 by C4b2a (the C3 convertase of the classic and lectin initiation pathways), as well as by C3bBb (the C3 convertase of the AP), pegcetacoplan reduces the formation and activation of both the C3 and C5 convertases (80). Hence, pegcetacoplan inhibits complement activity triggered by all 3 initiating pathways.

In the NOBLE trial, 13 kidney transplant patients with recurrent disease (10 C3G, 3 IC-MPGN) received pegcetacoplan (10 patients, 8 C3G) or standard-of-care only (3 patients, 2 C3G) for 12 weeks (81). The primary endpoint was reduction in C3 staining on repeat biopsy. At 12 weeks, 5 of 10 treated patients had ≥ 2 orders-of-magnitude reduction (OOM) in C3 staining, including 4 with 0 staining and no deposits on electron microscopy, and 8 of 10 had ≥ 1 OOM reduction; only 1 of 3 controls had reduction. There was a median 54% reduction in proteinuria in the 5 pegcetacoplan treated patients with UPCR ≥ 1g/g, and the C3G histology index acute score decreased in 8 of 10.

The phase 3 VALIANT trial randomized 124 patients with native kidney C3G, recurrent C3G, or native kidney IC-MPGN to pegcetacoplan or placebo for 26 weeks in a double-blind trial (82, 83). The primary endpoint was reduction in UPCR at 26 weeks. Pegcetacoplan-treated patients had a 68.1% relative reduction versus placebo (P<0.0001). The composite renal endpoint (≥ 50% reduction in UPCR with stable eGFR) occurred in 49% pegcetacoplan-treated patients versus 3% given placebo (P<0.0001). However, the C3G histologic index activity score did not significantly differ in the 69 with evaluable biopsies. Although not formally tested, the mean difference in eGFR at 26 weeks was 6.3 ml/min/1.73m^2^ and C3 staining on repeat biopsy was reduced. Pegcetacoplan (Empaveli^R^, Apellis Pharmaceuticals) is now FDA approved (July 28,2025) for adults and children > 12 years old for both primary IC-MPGN and C3G to reduce proteinuria.

Factor D activates Factor B and is the rate limiting step in the AP amplification loop. Inhibition of Factor D is a logical choice to suppress AP overactivity. The oral Factor D inhibitor danicopan was studied in 2 small phase 2 studies involving 35 patients (34 C3G, 1 IC-MPGN), one placebo controlled and one open-label (84). Unfortunately, in the dosages used, sufficient AP inhibition was not maintained as recovery occurred in a few hours. The primary endpoints, change from baseline to 6 months or 12 months in a composite biopsy score, were not met, nor was there a reduction in proteinuria of ≥ 30% by danicopan in the placebo-controlled trial (13 patients). In the open label trial (22 patients), proteinuria was reduced in about one-third. The studies were terminated early for futility.

The oral factor B inhibitor iptacopan does not stop spontaneous hydrolysis of C3 to C3(H_2_0). By binding to Factore B, iptacopan inhibits both formation of the AP C3 convertase C3bBb and, more importantly, its enzymatic activity. The result is reduced amplified C3b production. Thus, downstream activation of the alternate pathway C5 convertase (C3bBbC3b) is inhibited to reduce C5a and C5b-9 production. Activation of the C3 convertase of the classical and lectin pathways (C4b2a) is not inhibited, although amplification is.

An open-label phase 2 study of iptacopan in C3G involved 16 patients with native kidney disease and 11 transplant patients with recurrence treated for 12 weeks (85). The UPCR decreased by 45% (p=0.0003) at 12 weeks in the native kidney cohort, and C3 staining significantly decreased in the transplant cohort (p=0.03). In the 12-month extension study, the patients with native kidney disease maintained reduced UPCR (57%, p<0.0001) with improved eGFR (by 6.83 ml/min/1.73m^2^, p=0.0174) and the transplant patients maintained low proteinuria and stable eGFR. Both cohorts had significantly elevation in serum C3 levels (86).

The phase 3, double-blind, placebo-controlled APPEAR-C3G trial randomized 74 C3G patients with low serum C3, UPCR ≥ 1.0, and eGFR ≥ 30 from 18 countries to iptacopan 200 mg bid or placebo for 6 months followed by a 6-month open-label extension in which all patients received active drug (87). The primary end-point (between group relative UPCR reduction at 6-months) was significantly positive (35% reduction relative to placebo, p=0.0014) as was the secondary composite renal endpoint (≥ 50% reduction in UPCR with stable eGFR, 30% versus 6%, odds ratio 7.15, p=0.0166). There was no significant difference in GFR between groups at 6 months, although comparing eGFR slope for the whole population pre-iptacopan to post-iptacopan showed a change in slope of 9.01 ml/min/173m^2^/year (p<0.0001). Serum C3 significantly increased, and both serum and urine C5b-9 were significantly decreased (P¾ 0.0001 for all three). In comparing 6-month to baseline biopsies, glomerular C3 deposition was significantly reduced versus placebo (p=0.0053). Iptacopan (Fabhalta^R^, Novartis) has received FDA approval for treating adults with C3G to reduce proteinuria.

## Discussion

Primary MPGN, which includes both IC-MPGN and C3G, is an ultrarare glomerulopathy that requires a biopsy for diagnosis and is distinguished from other conditions having a membranoproliferative pattern on LM by bright C3 staining on immunofluorescence. However, C3 staining (typically against C3c) may be weak or even negative in some cases otherwise diagnosable as C3G, especially DDD (88). Staining for C3d may be positive in such cases (88). MPGN occurs at any age but is more common in children. The presentation is variable ranging from asymptomatic urinary sediment abnormalities with or without reduced eGFR to nephrotic syndrome to chronic nephritic syndrome to rapidly progressive, crescentic glomerulonephritis.

The approach to MPGN begins with exclusion of secondary causes, especially if immunosuppression is considered. For IC-MPGN, these consist of infections, autoimmune diseases, neoplasia, and monoclonal gammopathies. Chronic antigenic stimulation is the likely cause. The most common infections include chronic hepatitis (B and C, with or without cryoglobulins), chronic bacterial infections (endocarditis, ventriculoatrial shunt infections, abscesses), protozoal infections (malaria, schistosomiasis), and others (89). Autoimmune causes include systemic lupus, rheumatoid arthritis, primary Sjogren’s syndrome, primary sclerosing cholangitis, Graves’ disease, and others (90). Underlying cancer should be excluded by age-appropriate screening, given the 3-fold increased risk (91). B-cell lineage neoplasms have been associated, such as chronic lymphatic leukemia (92) via an associated monoclonal gammopathy, but solid tumors are reported as well (93). Monoclonal gammopathies, non-malignant or malignant, are found in 20 – 40% of MPGN cases (59, 94). Light chain restriction indicates monoclonality, but initial immunofluorescence may be equivocal, and monoclonality can be established with staining after pronase digestion of formalin-fixed, paraffin-embedded tissue (95) or by laser microdissection and mass spectrometry (96). Monoclonality indicates clone-directed therapy, as discussed above, and not immunosuppression.

Likewise, secondary causes of C3G must be excluded, most notably monoclonal gammopathies and infection-related glomerulonephritis (IRGN). Monoclonal proteins can act as autoantibodies, targeting regulatory proteins such as CFH (97), CFI, and CR1, and they may be C3-activating (98). As noted above, IC-MPGN associated with monoclonal proteins may have masked immunoglobulins and appear as C3G requiring pronase digestion or mass spectrometry to uncover the immunoglobulins. Clone-directed therapy should be implemented, if a monoclonal protein accompanies C3G, regardless of any monoclonality detectable on the biopsy. IRGN can completely mimic C3G histologically (99). Recent or ongoing infection, transient depression of C3 (resolves in weeks), spontaneous resolution of nephropathy, subepithelial humps on electron microscopy, moderate-to-strong glomerular C4d staining (100), anti-CFB autoantibodies in children (101), and staining for nephritis-associated plasmin receptor (NAPlr) with enhanced plasmin activity all together suggest IRGN (99).

The pathophysiology and prognosis for the 3 major subtypes of primary MPGN (IC-MPGN, C3GN, and DDD) are similar and patients can transition from one variant to another on serial biopsies. Hence, they are considered together. It remains possible that immune complexes are the initiating factor for IC-MPGN, but dysregulated AP activity nevertheless drives the process. Also, a similar proportion of IC-MPGN cases has both complement mutations and autoantibodies as in C3G.

In our opinion, upon diagnosis, complete genetic screening should be performed for the full battery of genes typically assessed for aHUS (or as a minimum *C3, CFB, CFH, CFI*), as pathogenic mutations are found in up to 20% of cases. Although defined mutations do not currently determine therapy nor define suitability for transplantation, the knowledge may become useful in the future as more is learned. Likewise, we recommend screening for autoantibodies (NeFs, anti-CFH, -CFI, -C3, and -CFB), as they are found in 40 – 80% of patients. Although not relevant in determining suitability for transplantation, when present they suggest a better chance for response to non-specific immunosuppression, such as MMF/corticosteroids, if being considered and anti-complement therapy is not available.

The prognosis is variable, with about 40 – 50% reaching KF at 10 years. All patients should have background therapy for other primary glomerulopathies, such as strict blood pressure control, RAS inhibition, SGLT2 inhibition, weight reduction if obese, etc. The questions of *who* to immunosuppress and *how* to remain to be better defined. There are obvious limitations to available data. Most studies are retrospective. The main limitations to evaluating therapy stem from the rarity of these diseases and the relatively long evolution requiring surrogate endpoints. Furthermore, the pathogenesis is multifactorial with genetic influences and autoantibodies potentially involved to differing degrees among patients; however, neither should determine immunosuppression or transplantation suitability.

At diagnosis, a reduced GFR for no other reason would be the worst prognostic factor in our opinion, along with declining GFR over time. In either circumstance we recommend immunosuppression. The relationship of baseline proteinuria to outcome has been inconsistent. However, proteinuria at one year, considered as an absolute amount or a percentage change from baseline, has consistently been shown to be a reasonably valid surrogate. An inflection point occurs at approximately one gram per day above which the risk for KF abruptly increases. Persisting proteinuria above this level should prompt consideration of immunosuppression.

Cluster analysis which can be assigned via on-line applicator (*vide supra*) is promising, but assignment to a specific cluster as a determinant of immunosuppression requires validation in other populations and preferably in RCTs. The simple scoring system of Chauvet et al. (53) also requires validation but intuitively seems a useful adjunct.

The ongoing development of complement inhibitors has changed the landscape of immunosuppression for primary MPGN. In general, we recommend immunosuppression when patients satisfy entry criteria for the APPEAR-C3G and VALIANT trials, i.e., persisting proteinuria > 1 gram/day (despite maximal supportive care) and eGFR > 30. Pegcetacoplan inhibits C3 and hence the AP at its genesis. Furthermore, C5 convertase activity of all 3 pathways would be reduced. Hence, this would seem the best choice for IC-MPGN, where both the CP (at least at initiation) and the AP (especially for propagation) would be involved. The impressive preliminary results of VALIANT support this contention, but it does require parenteral administration. Pegcetacoplan would be our first choice for immunosuppression of IC-MPGN.

The oral CFB inhibitor iptacopan has similarly shown impressive results in the APPEAR-C3G trial, while patients with IC-MPGN are being evaluated in a separate trial. In our opinion, either pegcetacoplan or iptacopan would be first line for immunosuppressing C3G, the former requiring twice weekly injections and the latter twice daily oral administration. They appear equally effective and safe based on available data. The CFD inhibitor danicopan, approved for PNH, should not be used given its inability to suppress AP activity completely at available doses.

Recurrence following transplantation clearly shortens allograft survival. In our opinion, protocol biopsies are indicated, perhaps at 3 months and one year. If C3 deposits are present, especially with concurrent electron-dense deposits, immunosuppression (complement inhibition) should be considered regardless of light microscopic changes or clinical manifestations. Data indicate progression is likely. Both iptacopan (85) and pegcetacoplan (81) have shown the ability to significantly reduce C3 staining on repeat biopsy with clinical improvement as well. Either should be strongly considered for early recurrence.

C5 inhibition with eculizumab or ravulizumab seems best suited for cases with AP dysregulation at the tissue level, such as aHUS, as opposed to a predominantly fluid phase disorder such as MPGN. The results with eculizumab in MPGN have been inconsistent, with observational data suggesting a possible benefit in rapidly progressive, crescentic cases (76). C3 overactivity, the presumed pathogenic driver would not be reduced. We prefer iptacopan or pegcetacoplan even for more aggressive cases given the positive data from RCTs for MPGN in general and the lack of such trials for eculizumab. It is unclear if elevated C5b-9 levels may indicate a better chance to respond to C5 inhibition versus CFB or C3.

The role of avacopan, if any, is unclear. The primary endpoint of ACCOLADE was negative, although there was a potential signal for reduced progression of fibrosis. It remains to be determined if cases with excessive inflammation on biopsy would be more likely to respond given the potent inflammatory effect of C5a. It also remains unclear if combining either C5 or C5a inhibition with earlier complement blockade will have increased efficacy with acceptable safety. No such combination trials are planned or in progress to our knowledge.

## Conclusion

Primary MPGN is an ultrarare group of diseases with variable presentation and clinical course (see **Table 4** for takeaway points). Perhaps half will progress to kidney failure with a high rate of recurrence following transplantation. Patients should be screened for paraproteins, the presence of which dictates clone directed therapy. Complement protein mutations and/or autoantibodies can be screened for and are found in most patients but should not dictate therapy or alter consideration for transplantation. If immunosuppression is considered, complement inhibition with pegcetacoplan for IC-MPGN and either iptacopan or pegcetacoplan for C3G should be first line. The role of C5 inhibition is unclear. If complement inhibition is unavailable, MMF/corticosteroids can be considered, especially if autoantibodies are present. Following transplantation, protocol biopsies should be performed routinely, with consideration of complement inhibition (iptacopan or pegcetacoplan) if C3 deposits are found regardless of LM findings.

**Table 4:** Primary MPGN – key takeaways.

• Primary MPGN is characterized by obvious glomerular staining for C3 with (IC-MPGN) or without (C3G, including C3GN and DDD) associated immunoglobulins • Secondary causes must be excluded, including infections, autoimmune disease, and neoplasia • Complement protein mutations are found in about 20% and autoantibodies in 40 – 80% with overlap • Neither mutations nor autoantibodies should determine immunosuppression or suitability for transplantation • Paraproteins indicate clone directed therapy • First line immunosuppression includes the C3 inhibitor pegcetacoplan (for IC-MPGN or C3G) or the Factor B inhibitor iptacopan (for C3G) • MMF/steroids can be considered if complement inhibitors are unavailable • Recurrence following transplantation is common and shortens allograft survival • Early surveillance biopsies following transplantation should be performed with intent of complement inhibition if C3 deposits are found.

C3G, C3 glomerulopathy; C3GN, C3 glomerulonephritis; DDD, dense deposit disease; IC-MPGN, immune complex membranoproliferative glomerulonephritis; MMF, mycophenolate mofetil; MPGN, membranoproliferative glomerulonephritis

## References

[1] Sethi S Fervenza FC . Membranoproliferative glomerulonephritis—a new look at an old entity. N Engl J Med. (2012) 366:1119–31. doi: 10.1056/NEJMra1108178, PMID: 22435371

[2] Madden B Singh RD Haas M Palma LMP Sharma A Vargas MJ . Apolipoprotein E is enriched in dense deposits and is a marker for dense deposit disease in C3 glomerulopathy. Kidney Int. (2024) 105:1077–87. doi: 10.1016/j.kint.2024.02.013, PMID: 38447879

[3] Hurdogan O Mirioglu S Dirim AB Doksan M Yuruk Yildirim ZN Turkmen A . Apolipoprotein E immunostaining has diagnostic utility in differentiating dense deposit disease and C3 glomerulonephritis: clone-based evaluation of D719N, EP1373Y, and 1B2C9. Modern Pathol. (2025) 38:100874. doi: 10.1016/j.modpat.2025.100874, PMID: 40865921

[4] Nakagawa N Hasebe N Hattori M Nagata M Yokoyama H Sato H . Clinical features and pathogenesis of membranoproliferative glomerulonephritis: a nationwide analysis of the Japan renal biopsy registry from 2007 to 2015. Clin Exp Nephrol. (2018) 22:797–807. doi: 10.1007/s10157-017-1513-7, PMID: 29214407

[5] Bernardes TP Mastroianni-Kirsztajn G . Membranoproliferative glomerulonephritis: current histopathological classification, clinical profile, and kidney outcomes. J Bras Nefrol. (2023) 45:45–50. doi: 10.1590/2175-8239-JBN-2022-0016en, PMID: 35789244 PMC10139719

[6] Dobronravov VA Smirnov AV . Membranoproliferative glomerulonephritis in Russian population. Ter Arkh. (2018) 90:39–47. doi: 10.26442/00403660.2018.12.000007, PMID: 30701832

[7] Bajeer IA Khatri S Kumar P Hashmi S Mubarak M Lanewala AA . Clinical characteristics and short term outcomes of childhood immune complex membranoproliferative glomerulonephritis and C3 glomerulopathy: a single centre retrospective study. BMC Nephrol. (2025) 26:143–3. doi: 10.1186/s12882-025-04078-3, PMID: 40121417 PMC11929162

[8] Schmitt H Bohle A Reineke T Mayer-Eichberger D Vogl W . Long-term prognosis of membranoproliferative glomerulonephritis type I: significance of clinical and morphological parameters: an investigation of 220 cases. Nephron. (2008) 55:242–50. doi: 10.1159/000185969, PMID: 2370922

[9] Schaefer F Hofstetter J Ruiz EM Antonucci L Conlon PJ Francesca B . 1196 C3G and ic-MPGN across the life span: findings from the European Rare Kidney Disease Registry. Nephrol Dial Transplant. (2024) 39:gfae069–1196. doi: 10.1093/ndt/gfae069.029

[10] Masoud S Wong K Pitcher D Downward L Proudfoot C Webb NJA . Quantifying association of early proteinuria and estimated glomerular filtration rate changes with long-term kidney failure in C3 glomerulopathy and immune-complex membranoproliferative glomerulonephritis using the United Kingdom RaDaR Registry. Kidney Int. (2025) 108:455–69. doi: 10.1016/j.kint.2025.06.003, PMID: 40582408

[11] Kirpalani A Jawa N Smoyer WE Licht C Midwest Pediatric Nephrology Consortium . Long-term outcomes of C3 glomerulopathy and immune-complex membranoproliferative glomerulonephritis in children. Kidney Int Rep. (2020) 5:2313–24. doi: 10.1016/j.ekir.2020.09.019, PMID: 33305125 PMC7710848

[12] Kim S Choi YE Kim SG Ryu D Park S Koo TY . Clinical features and outcomes of immune complex-membranoproliferative glomerulonephritis and C3 glomerulopathy: a multicenter observational cohort study analyzing kidney biopsy cases. Kidney Res Clin Pract. (2024). doi: 10.23876/j.krcp.24.129, PMID: 39980095

[13] Lemaire M Noone D Lapeyraque A Licht C Frémeaux-Bacchi V . Inherited kidney complement diseases. Clin J Am Soc Nephrol. (2021) 16. doi: 10.2215/CJN.11830720, PMID: 33536243 PMC8216622

[14] Forneris F Ricklin D Wu J Tzekou A Wallace RS Lambris JD . Structures of C3b in complex with factors B and D give insight into complement convertase formation. Science. (2010) 330:1816–20. doi: 10.1126/science.1195821, PMID: 21205667 PMC3087196

[15] Piras R Breno M Valoti E Alberti M Iatropoulos P Mele C . CFH and CFHR copy number variations in C3 glomerulopathy and immune complex-mediated membranoproliferative glomerulonephritis. Front Genet. (2021) 12:670727. doi: 10.3389/fgene.2021.670727, PMID: 34211499 PMC8240960

[16] Tortajada A Yébenes H Abarrategui-Garrido C Anter J García-Fernández JM Martínez-Barricarte R . C3 glomerulopathy-associated CFHR1 mutation alters FHR oligomerization and complement regulation. J Clin Invest. (2013) 123:2434–46. doi: 10.1172/JCI68280, PMID: 23728178 PMC3668852

[17] Frangou E Varnavidou-Nicolaidou A Petousis P Soloukides A Theophanous E Savva I . Clinical course and outcome after kidney transplantation in patients with C3 glomerulonephritis due to CFHR5 nephropathy. Nephrol Dialysis Transplant. (2019) 34:1780–8. doi: 10.1093/ndt/gfz021, PMID: 30844074

[18] Liszewski MK Atkinson JP . Complement regulator CD46: genetic variants and disease associations. Hum Genomics (2015) 9:7. doi: 10.1186/s40246-015-0029-z, PMID: 26054645 PMC4469999

[19] Brocklebank V Kumar G Howie AJ Chandar J Milford DV Craze J . Long-term outcomes and response to treatment in diacylglycerol kinase epsilon nephropathy. Kidney Int. (2020) 97:1260–74. doi: 10.1016/j.kint.2020.01.045, PMID: 32386968 PMC7242908

[20] Servais A Noël L Roumenina LT Le Quintrec M Ngo S Dragon-Durey M . Acquired and genetic complement abnormalities play a critical role in dense deposit disease and other C3 glomerulopathies. Kidney Int. (2012) 82:454–64. doi: 10.1038/ki.2012.63, PMID: 22456601

[21] Iatropoulos P Noris M Mele C Piras R Valoti E Bresin E . Complement gene variants determine the risk of immunoglobulin-associated MPGN and C3 glomerulopathy and predict long-term renal outcome. Mol Immunol. (2016) 71:131–42. doi: 10.1016/j.molimm.2016.01.010, PMID: 26895476

[22] Bu F Borsa NG Jones MB Takanami E Nishimura C Hauer JJ . High-throughput genetic testing for thrombotic microangiopathies and C3 glomerulopathies. J Am Soc Nephrol. (2016) 27:1245–1253. doi: 10.1681/ASN.2015040385, PMID: 26283675 PMC4814193

[23] Meuleman MS Vieira-Martins P El Sissy C Audard V Baudouin V Bertrand D . Rare variants in complement gene in C3 glomerulopathy and immunoglobulin-mediated membranoproliferative GN. Clin J Am Soc Nephrol. (2023) 18:1435–1445. doi: 10.2215/CJN.0000000000000252, PMID: 37615951 PMC10637453

[24] Levine AP Chan MM Sadeghi-Alavijeh O Wong EK Cook HT Ashford S . Large-scale whole-genome sequencing reveals the genetic architecture of primary membranoproliferative GN and C3 glomerulopathy. J Am Soc Nephrol. (2020) 31:365–73. doi: 10.1681/ASN.2019040433, PMID: 31919107 PMC7003307

[25] Vivarelli M Barratt J Beck LH Fakhouri F Gale DP Goicoechea de Jorge E . The role of complement in kidney disease: conclusions from a Kidney Disease: Improving Global Outcomes (KDIGO) Controversies Conference. Kidney Int. (2024) 106:369–91. doi: 10.1016/j.kint.2024.05.015, PMID: 38844295

[26] Heurich M Martínez-Barricarte R Francis NJ Roberts DL Rodríguez de Córdoba S Morgan BP . Common polymorphisms in C3, factor B, and factor H collaborate to determine systemic complement activity and disease risk. Proc Natl Acad Sci U.S.A. (2011) 108:8761–6. doi: 10.1073/pnas.1019338108, PMID: 21555552 PMC3102398

[27] Ding Y Zhao W Zhang T Qiang H Lu J Su X . A haplotype in CFH family genes confers high risk of rare glomerular nephropathies. Sci Rep. (2017) 7:6004. doi: 10.1038/s41598-017-05173-8, PMID: 28729648 PMC5519609

[28] Corvillo F Okrój M Nozal P Melgosa M Sánchez-Corral P López-Trascasa M . Nephritic factors: an overview of classification, diagnostic tools and clinical associations. Front Immunol. (2019) 10:886. doi: 10.3389/fimmu.2019.00886, PMID: 31068950 PMC6491685

[29] Michels MAHM Wijnsma KL Kurvers RAJ Westra D Schreuder MF van Wijk JAE . Long-term follow-up including extensive complement analysis of a pediatric C3 glomerulopathy cohort. Pediatr Nephrol. (2022) 37:601–12. doi: 10.1007/s00467-021-05221-6, PMID: 34476601 PMC8921070

[30] Garam N Prohászka Z Szilágyi Á Aigner C Schmidt A Gaggl M . C4 nephritic factor in patients with immune-complex-mediated membranoproliferative glomerulonephritis and C3-glomerulopathy. Orphanet J Rare Dis. (2019) 14:247–8. doi: 10.1186/s13023-019-1237-8, PMID: 31703608 PMC6839100

[31] Marinozzi M Chauvet S Le Quintrec M Mignotet M Petitprez F Legendre C . C5 nephritic factors drive the biological phenotype of C3 glomerulopathies. Kidney Int. (2017) 92:1232–41. doi: 10.1016/j.kint.2017.04.017, PMID: 28712854

[32] Zhang Y Ghiringhelli Borsa N Shao D Dopler A Jones MB Meyer NC . Factor H autoantibodies and complement-mediated diseases. Front Immunol. (2020) 11:607211. doi: 10.3389/fimmu.2020.607211, PMID: 33384694 PMC7770156

[33] Li L Li Z Wang S Yu X Tan Y Wang Y . Monoclonal immunoglobulin mediates complement activation in monoclonal gammopathy associated-C3 glomerulonephritis. BMC Nephrol. (2019) 20:459–3. doi: 10.1186/s12882-019-1640-3, PMID: 31823738 PMC6902416

[34] Marinozzi MC Roumenina LT Chauvet S Hertig A Bertrand D Olagne J . Anti-factor B and anti-C3b autoantibodies in C3 glomerulopathy and Ig-associated membranoproliferative GN. J Am Soc Nephrol. (2017) 28:1603–13. doi: 10.1681/ASN.2016030343, PMID: 28096309 PMC5407719

[35] Andresdottir MB Assmann KJ Hoitsma AJ Koene RA Wetzels JF . Renal transplantation in patients with dense deposit disease: morphological characteristics of recurrent disease and clinical outcome. Nephrology dialysis Transplant. (1999) 14:1723–31. doi: 10.1093/ndt/14.7.1723, PMID: 10435883

[36] Zand L Lorenz EC Cosio FG Fervenza FC Nasr SH Gandhi MJ . Clinical findings, pathology, and outcomes of C3GN after kidney transplantation. J Am Soc Nephrol. (2014) 25:1110–7. doi: 10.1681/ASN.2013070715, PMID: 24357668 PMC4005307

[37] Alasfar S Carter-Monroe N Rosenberg AZ Montgomery RA Alachkar N . Membranoproliferative glomerulonephritis recurrence after kidney transplantation: using the new classification. BMC Nephrol. (2016) 17:7–x. doi: 10.1186/s12882-015-0219-x, PMID: 26754737 PMC4709883

[38] Regunathan-Shenk R Avasare RS Ahn W Canetta PA Cohen DJ Appel GB . Kidney transplantation in C3 glomerulopathy: a case series. Am J Kidney Dis. (2019) 73:316–23. doi: 10.1053/j.ajkd.2018.09.002, PMID: 30413277

[39] Caravaca-Fontán F Polanco N Villacorta B Buxeda A Coca A Ávila A . Recurrence of immune complex and complement-mediated membranoproliferative glomerulonephritis in kidney transplantation. Nephrol Dialysis Transplant. (2023) 38:222–35. doi: 10.1093/ndt/gfac148, PMID: 35404425

[40] Tarragón B Peleg Y Jagannathan G Sekulic M Chang J Cohen DJ . C3 Glomerulopathy Recurs Early after Kidney Transplantation in Serial Biopsies Performed within the First 2 Years after Transplantation. Clin J Am Soc Nephrol. (2024) 19:1005–1015. doi: 10.2215/CJN.0000000000000474, PMID: 39116277 PMC11321730

[41] Halfon M Taffé P Bucher C Haidar F Huynh-do U Mani L . Outcome of patients transplanted for C3 glomerulopathy and primary immune complex-mediated membranoproliferative glomerulonephritis. Kidney Int Rep. (2024) 10:75–86. doi: 10.1016/j.ekir.2024.10.008, PMID: 39810762 PMC11725970

[42] Ames EG Anand PM Bekheirnia MR Doshi MD El Ters M Freese ME . Evaluation for genetic disease in kidney transplant candidates: A practice resource. Am J Transplant. (2025) 25:237–49. doi: 10.1016/j.ajt.2024.10.019, PMID: 39488252 PMC13266825

[43] Attieh RM Bharati J Sharma P Nair G Ayehu G Jhaveri KD . Kidney transplant in patients with C3 glomerulopathy. Clin Kidney J. (2025) 18:sfaf134. doi: 10.1093/ckj/sfaf134, PMID: 40385590 PMC12082085

[44] Medjeral-Thomas NR O’Shaughnessy MM O’Regan JA Traynor C Flanagan M Wong L . C3 glomerulopathy: clinicopathologic features and predictors of outcome. Clin J Am Soc Nephrol. (2014) 9:46–53. doi: 10.2215/CJN.04700513, PMID: 24178974 PMC3878702

[45] Bomback AS Santoriello D Avasare RS Regunathan-Shenk R Canetta PA Ahn W . C3 glomerulonephritis and dense deposit disease share a similar disease course in a large United States cohort of patients with C3 glomerulopathy. Kidney Int. (2018) 93:977–85. doi: 10.1016/j.kint.2017.10.022, PMID: 29310824

[46] Kovala M Seppälä M Räisänen-Sokolowski A Meri S Honkanen E Kaartinen K . Diagnostic and prognostic comparison of immune-complex-mediated membranoproliferative glomerulonephritis and C3 glomerulopathy. Cells. (2023) 12:712. doi: 10.3390/cells12050712, PMID: 36899849 PMC10000503

[47] Ghaddar M Caravaca-Fontán F Praga M Fernández-Juárez G Lomax-Browne H Cook HT . Clinical and histologic predictors of kidney outcomes in C3 glomerulopathy and idiopathic membranoproliferative GN. Clin J Am Soc Nephrol. (2025) 20:1119–1131. doi: 10.2215/CJN.0000000751, PMID: 40512548 PMC12342078

[48] Caliskan Y Torun ES Tiryaki TO Oruc A Ozluk Y Akgul SU . Immunosuppressive treatment in C3 glomerulopathy: is it really effective? Am J Nephrol. (2017) 46:96–107. doi: 10.1159/000479012, PMID: 28700996

[49] Ravindran A Fervenza FC Smith RJH De Vriese AS Sethi S . C3 glomerulopathy: ten years’ Experience at mayo clinic. Mayo Clinic Proc. (2018) 93:991–1008. doi: 10.1016/j.mayocp.2018.05.019, PMID: 30077216 PMC6312642

[50] Avasare RS Canetta PA Bomback AS Marasa M Caliskan Y Ozluk Y . Mycophenolate mofetil in combination with steroids for treatment of C3 glomerulopathy: A case series. Clin J Am Soc Nephrol. (2018) 13:406–413. doi: 10.2215/CJN.09080817, PMID: 29326307 PMC5967675

[51] Caravaca-Fontán F Díaz-Encarnación MM Lucientes L Cavero T Cabello V Ariceta G . Mycophenolate mofetil in C3 glomerulopathy and pathogenic drivers of the disease. Clin J Am Soc Nephrol. (2020) 15:1287–1298. doi: 10.2215/CJN.15241219, PMID: 32816888 PMC7480558

[52] Caravaca-Fontán F Díaz-Encarnación M Cabello V Ariceta G Quintana LF Marco H . Longitudinal change in proteinuria and kidney outcomes in C3 glomerulopathy. Nephrol Dial Transplant. (2022) 37:1270–80. doi: 10.1093/ndt/gfab075, PMID: 33779754

[53] Chauvet S Hauer JJ Petitprez F Rabant M Martins PV Baudouin V . Results from a nationwide retrospective cohort measure the impact of C3 and soluble C5b-9 levels on kidney outcomes in C3 glomerulopathy. Kidney Int. (2022) 102:904–16. doi: 10.1016/j.kint.2022.05.027, PMID: 35752323 PMC10588728

[54] Iatropoulos P Daina E Curreri M Piras R Valoti E Mele C . Cluster analysis identifies distinct pathogenetic patterns in C3 glomerulopathies/immune complex–mediated membranoproliferative GN. J Am Soc Nephrol. (2018) 29:283–294. doi: 10.1681/ASN.2017030258, PMID: 29030465 PMC5748907

[55] Garam Nó Prohászka Z Szilágyi Á Aigner C Schmidt A Gaggl M . Validation of distinct pathogenic patterns in a cohort of membranoproliferative glomerulonephritis patients by cluster analysis. Clin Kidney J. (2020) 13:225–34. doi: 10.1093/ckj/sfz073, PMID: 32296528 PMC7147314

[56] Benigni A Daina E Löffler-Wirth H Piras R Rigoldi M Schmidt M . Hierarchical clustering uncovered disease patterns and further untangled complexities in immune complex-mediated idiopathic MPGN and C3 glomerulopathy. Kidney Int. (2025). doi: 10.1016/j.kint.2025.08.035, PMID: 41076080

[57] Rovin BH Adler SG Barratt J Bridoux F Burdge KA Chan TM . Executive summary of the KDIGO 2021 guideline for the management of glomerular diseases. Kidney Int. (2021) 100:753–79. doi: 10.1016/j.kint.2021.05.015, PMID: 34556300

[58] Bridoux F Javaugue V Nasr SH Leung N . Proliferative glomerulonephritis with monoclonal immunoglobulin deposits: a nephrologist perspective. Nephrol Dial Transplant. (2021) 36:208–15. doi: 10.1093/ndt/gfz176, PMID: 33494099

[59] Sethi S Zand L Leung N Smith RJH Jevremonic D Herrmann SS . Membranoproliferative glomerulonephritis secondary to monoclonal gammopathy. Clin J Am Soc Nephrol. (2010) 5:770–82. doi: 10.2215/CJN.06760909, PMID: 20185597 PMC2863981

[60] Chauvet S Frémeaux-Bacchi V Petitprez F Karras A Daniel L Burtey S . Treatment of B-cell disorder improves renal outcome of patients with monoclonal gammopathy–associated C3 glomerulopathy. Blood. (2017) 129:1437–47. doi: 10.1182/blood-2016-08-737163, PMID: 28069603

[61] Jones G Juszczak M Kingdon E Harber M Sweny P Burns A . Treatment of idiopathic membranoproliferative glomerulonephritis with mycophenolate mofetil and steroids. Nephrol Dial Transplant. (2004) 19:3160–4. doi: 10.1093/ndt/gfh526, PMID: 15479745

[62] Yuan M Zou J Zhang X Liu H Teng J Zhong Y . Combination therapy with mycophenolate mofetil and prednisone in steroid-resistant idiopathic membranoproliferative glomerulonephritis. Clin Nephrol. (2010) 73:354–9. doi: 10.5414/cnp73354, PMID: 20420795

[63] Rabasco C Cavero T Román E Rojas-Rivera J Olea T Espinosa M . Effectiveness of mycophenolate mofetil in C3 glomerulonephritis. Kidney Int. (2015) 88:1153–60. doi: 10.1038/ki.2015.227, PMID: 26221755

[64] Bharati J Tiewsoh K Kumar A Nada R Rathi M Gupta KL . Usefulness of mycophenolate mofetil in Indian patients with C3 glomerulopathy. Clin Kidney J. (2018) 12:483–7. doi: 10.1093/ckj/sfy127, PMID: 31384438 PMC6671524

[65] Yeter HH Sütiçen E Korucu B Helvaci Ö Özbaş B Gönül İ . Evaluation of clinical, laboratory and treatment modalities in C3 glomerulopathy: single center experience. Pril (Makedon Akad Nauk Umet Odd Med Nauki). (2019) 40:15–23. doi: 10.2478/prilozi-2019-0010, PMID: 31605593

[66] Khandelwal P Bhardwaj S Singh G Sinha A Hari P Bagga A . Therapy and outcomes of C3 glomerulopathy and immune-complex membranoproliferative glomerulonephritis. Pediatr Nephrol. (2021) 36:591–600. doi: 10.1007/s00467-020-04736-8, PMID: 32886193

[67] Pınarbaşı AS Dursun I Gokce I Çomak E Saygılı S Bayram MT . Predictors of poor kidney outcome in children with C3 glomerulopathy. Pediatr Nephrol. (2021) 36:1195–205. doi: 10.1007/s00467-020-04799-7, PMID: 33130981

[68] Rudnicki M . Rituximab for treatment of membranoproliferative glomerulonephritis and C3 glomerulopathies. BioMed Res Int. (2017) 2017:2180508. doi: 10.1155/2017/2180508, PMID: 28573137 PMC5440792

[69] van Schaik M de Vries APJ Bemelman FJ Rabelink TJ Trouw LA van Kooten C . Clinical remission and reduction of circulating nephritic factors by combining rituximab with belimumab in a case of complement factor 3 glomerulopathy. Kidney Int Rep. (2024) 9:1919–22. doi: 10.1016/j.ekir.2024.02.1402, PMID: 38899188 PMC11184254

[70] Nester C Decker DA Meier M Aslam S Bomback AS Caravaca-Fontán F . Developing therapies for C3 glomerulopathy: report of the kidney health initiative C3 glomerulopathy trial endpoints work group. Clin J Am Soc Nephrol. (2024) 19:1201–1208. doi: 10.2215/CJN.0000000000000505, PMID: 38829708 PMC11390019

[71] Vivarelli M Pasini A Emma F . Eculizumab for the treatment of dense-deposit disease. N Engl J Med. (2012) 366:1163–5. doi: 10.1056/NEJMc1111953, PMID: 22435383

[72] Daina E Noris M Remuzzi G . Eculizumab in a patient with dense-deposit disease. N Engl J Med. (2012) 366:1161–3. doi: 10.1056/NEJMc1112273, PMID: 22435382

[73] Radhakrishnan S Lunn A Kirschfink M Thorner P Hebert D Langlois V . Eculizumab and refractory membranoproliferative glomerulonephritis. N Engl J Med. (2012) 366:1165–6. doi: 10.1056/NEJMc1106619, PMID: 22435384

[74] Noris M Remuzzi G . C3G and ig-MPGN-treatment standard. Nephrol Dial Transplant. (2024) 39:202–14. doi: 10.1093/ndt/gfad182, PMID: 37604793 PMC10828209

[75] Bomback AS Smith RJ Barile GR Zhang Y Heher EC Herlitz L . Eculizumab for dense deposit disease and C3 glomerulonephritis. Clin J Am Soc Nephrol. (2012) 7:748–56. doi: 10.2215/CJN.12901211, PMID: 22403278 PMC3338285

[76] Le Quintrec M Lapeyraque A Lionet A Sellier-Leclerc A Delmas Y Baudouin V . Patterns of clinical response to eculizumab in patients with C3 glomerulopathy. Am J Kidney Dis. (2018) 72:84–92. doi: 10.1053/j.ajkd.2017.11.019, PMID: 29429752

[77] Ruggenenti P Daina E Gennarini A Carrara C Gamba S Noris M . C5 convertase blockade in membranoproliferative glomerulonephritis: A single-arm clinical trial. Am J Kidney Dis. (2019) 74:224–38. doi: 10.1053/j.ajkd.2018.12.046, PMID: 30929851

[78] Pickering MC Warren J Rose KL Carlucci F Wang Y Walport MJ . Prevention of C5 activation ameliorates spontaneous and experimental glomerulonephritis in factor H-deficient mice. Proc Natl Acad Sci. (2006) 103:9649–54. doi: 10.1073/pnas.0601094103, PMID: 16769899 PMC1476693

[79] Bomback AS Herlitz LC Kedia PP Petersen J Yue H Lafayette RA . Safety and efficacy of avacopan in patients with complement 3 glomerulopathy: randomized, double-blind clinical trial. J Am Soc Nephrol. (2025) 36:487–499. doi: 10.1681/ASN.0000000526, PMID: 39392695 PMC11888959

[80] Hillmen P Horneff R Yeh M Kolev M Deschatelets P . Navigating the complement pathway to optimize PNH treatment with pegcetacoplan and other currently approved complement inhibitors. Int J Mol Sci. (2024) 25. doi: 10.3390/ijms25179477, PMID: 39273426 PMC11395449

[81] Bomback AS Daina E Remuzzi G Kanellis J Kavanagh D Pickering MC . Efficacy and safety of pegcetacoplan in kidney transplant recipients with recurrent complement 3 glomerulopathy or primary immune complex membranoproliferative glomerulonephritis. Kidney Int Rep. (2025) 10:87–98. doi: 10.1016/j.ekir.2024.09.030, PMID: 39810766 PMC11725963

[82] Nester CM Bomback AS Ariceta Iraola MG Delmas Y Dixon BP Gale DP . VALIANT: A randomized, multicenter, double-blind, placebo (PBO)-controlled, phase 3 trial of pegcetacoplan for patients with native or post-transplant recurrent glomerulopathy (C3G) or primary immune complex membranoproliferative glomerulonephritis (IC-MPGN): SA-OR92. J Am Soc Nephrol. (2024) 35. doi: 10.1681/ASN.2024qdwvz5bg

[83] Fakhouri F Bomback AS Ariceta G Delmas Y Dixon BP Gale DP . Trial of pegcetacoplan in C3 glomerulopathy and immune-complex MPGN. N Engl J Med. (2025) 393:2210–20. doi: 10.1056/NEJMoa2501510, PMID: 41337715

[84] Nester C Appel GB Bomback AS Bouman KP Cook HT Daina E . Clinical outcomes of patients with C3G or IC-MPGN treated with the factor D inhibitor danicopan: final results from two phase 2 studies. Am J Nephrol. (2022) 53:687–700. doi: 10.1159/000527167, PMID: 36423588

[85] Wong E Nester C Cavero T Karras A Le Quintrec M Lightstone L . Efficacy and safety of iptacopan in patients with C3 glomerulopathy. Kidney Int Rep. (2023) 8:2754–64. doi: 10.1016/j.ekir.2023.09.017, PMID: 38106570 PMC10719607

[86] Nester CM Eisenberger U Karras A Le Quintrec M Lightstone L Praga M . Iptacopan reduces proteinuria and stabilizes kidney function in C3 glomerulopathy. Kidney Int Rep. (2025) 10:432–46. doi: 10.1016/j.ekir.2024.10.023, PMID: 39990880 PMC11843281

[87] Kavanagh D Bomback AS Vivarelli M Nester CM Remuzzi G Zhao M . Oral iptacopan therapy in patients with C3 glomerulopathy: a randomised, double-blind, parallel group, multicentre, placebo-controlled, phase 3 study. Lancet. (2025) 406:1587–98. doi: 10.1016/S0140-6736(25)01148-1, PMID: 41016405

[88] Snijders MLH van de Wall-Neecke BJ Hesselink DA Becker JU Clahsen-van Groningen MC . Utility of immunohistochemistry with C3d in C3 glomerulopathy. Mod Pathol. (2020) 33:431–9. doi: 10.1038/s41379-019-0348-z, PMID: 31477814

[89] Sethi S Nester CM Smith RJH . Membranoproliferative glomerulonephritis and C3 glomerulopathy: resolving the confusion. Kidney Int. (2012) 81:434–41. doi: 10.1038/ki.2011.399, PMID: 22157657 PMC4428602

[90] Zand L Fervenza FC Nasr SH Sethi S . Membranoproliferative glomerulonephritis associated with autoimmune diseases. J Nephrol. (2014) 27:165–71. doi: 10.1007/s40620-014-0049-0, PMID: 24500888

[91] Heaf JG Hansen A Laier GH . Quantification of cancer risk in glomerulonephritis. BMC Nephrol. (2018) 19:27. doi: 10.1186/s12882-018-0828-2, PMID: 29394927 PMC5797419

[92] Strati P Nasr SH Leung N Hanson CA Chaffee KG Schwager SM . Renal complications in chronic lymphocytic leukemia and monoclonal B-cell lymphocytosis: the Mayo Clinic experience. Haematologica. (2015) 100:1180–8. doi: 10.3324/haematol.2015.128793, PMID: 26088927 PMC4800708

[93] Jhaveri KD Shah HH Calderon K Campenot ES Radhakrishnan J . Glomerular diseases seen with cancer and chemotherapy: a narrative review. Kidney Int. (2013) 84:34–44. doi: 10.1038/ki.2012.484, PMID: 23364518

[94] Kovala M Seppala M Kaartinen K Meri S Honkanen EO Räisänen-Sokolowski A . Mo085monoclonal gammopathy of unknown significance among membranoproliferative glomerulonephritis patients. Nephrol Dial Transplant. (2021) 36:gfab078.0021. doi: 10.1093/ndt/gfab078.0021

[95] Larsen CP Messias NC Walker PD Fidler ME Cornell LD Hernandez LH . Membranoproliferative glomerulonephritis with masked monotypic immunoglobulin deposits. Kidney Int. (2015) 88:867–73. doi: 10.1038/ki.2015.195, PMID: 26154922 PMC4687465

[96] Jain D Green JA Bastacky S Theis JD Sethi S . Membranoproliferative glomerulonephritis: the role for laser microdissection and mass spectrometry. Am J Kidney Dis. (2014) 63:324–8. doi: 10.1053/j.ajkd.2013.09.007, PMID: 24145022

[97] Johnson CK Zuniga SC Dhawale T Zhang Y Smith RJH Blosser CD . Monoclonal gammopathy of renal significance causes C3 glomerulonephritis via monoclonal igG kappa inhibition of complement factor H. Kidney Int Rep. (2021) 6:2505–9. doi: 10.1016/j.ekir.2021.06.015, PMID: 34514215 PMC8418982

[98] Chauvet S Roumenina LT Aucouturier P Marinozzi M Dragon-Durey M Karras A . Both monoclonal and polyclonal immunoglobulin contingents mediate complement activation in monoclonal gammopathy associated-C3 glomerulopathy. Front Immunol. (2018) 9:2260. doi: 10.3389/fimmu.2018.02260, PMID: 30333829 PMC6175995

[99] Wada Y Kamata M Miyasaka R Abe T Kawamura S Takeuchi K . Clinico-pathogenic similarities and differences between infection-related glomerulonephritis and C3 glomerulopathy. Int J Mol Sci. (2023) 24:8432. doi: 10.3390/ijms24098432, PMID: 37176142 PMC10179079

[100] Bashir S Hussain M Afzal A Hassan U Hameed M Mushtaq S . C4d at crossroads between post-infectious glomerulonephritis and C3 glomerulopathy. Int J Nephrol Renovasc Dis. (2021) 14:87–95. doi: 10.2147/IJNRD.S285302, PMID: 33732010 PMC7958999

[101] Chauvet S Berthaud R Devriese M Mignotet M Vieira Martins P Robe-Rybkine T . Anti-factor B antibodies and acute postinfectious GN in children. J Am Soc Nephrol. (2020) 31:829–840. doi: 10.1681/ASN.2019080851, PMID: 32034108 PMC7191928

[102] Zipfel PF Wiech T Stea ED Skerka C . CFHR gene variations provide insights in the pathogenesis of the kidney diseases atypical hemolytic uremic syndrome and C3 glomerulopathy. J Am Soc Nephrol. (2020) 31:241–56. doi: 10.1681/ASN.2019050515, PMID: 31980588 PMC7003313

[103] Fei Z Zhen K Zhang F Liu P Xu H Chen H . Clinical research advances of CFHR5 nephropathy: a recent review. Eur Rev Med Pharmacol Sci. (2023) 27:9987–10000. doi: 10.26355/eurrev_202310_34179, PMID: 37916369

[104] Garam N Cserhalmi M Prohászka Z Szilágyi Á Veszeli N Szabó E . FHR-5 serum levels and CFHR5 genetic variations in patients with immune complex-mediated membranoproliferative glomerulonephritis and C3-glomerulopathy. Front Immunol. (2021) 12:720183. doi: 10.3389/fimmu.2021.720183, PMID: 34566977 PMC8461307

[105] Thurman JM Fremeaux-Bacchi V . Alternative pathway diagnostics. Immunol Rev. (2023) 313:225–38. doi: 10.1111/imr.13156, PMID: 36305168 PMC9851998

